# Resections—Military Surgery

**Published:** 1863-05

**Authors:** Mord. Brooks

**Affiliations:** Asst. Surg. 82d Reg. Ind. Vols.


### ORIGINAL COMMUNICATIONS.

### RESECTIONS—MILITARY SURGERY.

By MORD. BROOKS, Asst. Surg. 82d Regt. Ind. Vols

At the General Field Hospital, shortly after the battle of Stone River, I happened to be a "looker on in Venice," when an incident like the following occurred:

A certain surgeon, who enjoyed the confidence of the Medical Director of the Department, and doubtless deserved it, had received permission to select such cases for operation as he might think of peculiar interest or difficulty.

A patient was placed on the table: it was a case of gunshot fracture of the Tibia The ball—a conical musket ball—had taken effect at the anterior aspect of the bone, about its lower third. On examination, our surgeon decided that it was a case for resection; at which time, the surgeon of the regiment to which the patient belonged appeared, and objected. The Brigade Surgeon was counseled: he said there might be some small spiculæ of bone which *should* be removed without enlarging the wound. Our surgeon declared it was necessary to enlarge it an inch each way. On appealing to the Medical Director the surgeon was ordered to proceed on his own judgment. The wound was enlarged and about fifty fragments of bone were taken out, one piece as large as the index finger. The operation was an enlightener to some concerned.

Being myself detailed at the same time as an assistant at one of the field hospitals, I was reminded by this circumstance to give more attention to the subject of resection.

A case recurs to my mind, which further illustrates what appears to me to be a fact, that army surgeons in general are not aware of the great amount of comminution that almost invariably attends these fractures, when made by the Minie ball.

This patient, like the one preceding, had received a gunshot fracture of the Tibia. The wound was examined and dressed by intelligent surgeons. Resection was not deemed advisable; it being supposed that there was not comminution sufficient to warrant its propriety.

Gangrene supervening about the fifteenth day, amputation was performed. On examining the limb, I was astonished at what was presented. It seemed more like a case of crushing. A number of spiculæ and several large pieces of bone were detached, and the shaft of the Tibia was split down to the Tarsal articulation.

Examination of the limb, after amputation, in another case of a similar character, revealed the same condition.

Whether even resection would have saved the limbs of these men or not, is an interesting question, that cannot, positively, be answered in the affirmative. I think, however, it would be in one instance. One thing I am certain of, they are a sample of a large number of cases in which secondary amputation has been necessary, and, in most instances, death the result.

On the whole, I am inclined to the opinion, that a gunshot fracture of limb, should almost in no instance be bound up without resection.

---

Microscopical Examination of the Air.—A series of experiments, originated by M. Revial at the Hospital Lariboisiere has shown the experience of a large amount of organic matter floating in the air. The dust collected from one of the wards contained thirty-six per cent., chiefly composing epithelial cells exhaling the smell of calcined horn or bone. In the air of those rooms where there are sufferers from contagious inflammation of the eyeball, small corpuscles were detached by the microscope, analogous to the virus thrown off by the inflamed eye. It is, therefore, to be assumed, on evidence strictly logical, if not absolute, that the infection is mechanically conveyed by the air.

